# Risk-based redesign of internal quality control procedures for emergency immunoassays in clinical laboratories: a multicenter study from China

**DOI:** 10.3389/fmed.2026.1906788

**Published:** 2026-07-27

**Authors:** Guangjun Xiao, Juan Hu, Yanting Liu, Sihan Zhao, Weifeng Liao, Huanhuan Wang, Shaocheng Zhang

**Affiliations:** 1 Department of Clinical Laboratory, Suining Central Hospital, Suining, Sichuan, China; 2 School of Laboratory Medicine, Chengdu Medical College, Chengdu, Sichuan, China; 3 Clinical IVD Joint Research Center of Chengdu Medical College-Maccura Biotechnology, Chengdu, Sichuan, China; 4 Department of Laboratory Medicine, The Second Affiliated Hospital of Chengdu Medical College (Nuclear Industry 416 Hospital), Chengdu, Sichuan, China; 5 Irradiation Preservation and Effect Key Laboratory of Sichuan, Chengdu, Sichuan, China

**Keywords:** emergency immunoassays, internal quality control, risk-based quality control, sigma metrics, Westgard rules

## Abstract

**Background:**

Although clinical laboratories routinely implement internal quality control (IQC) to ensure the reliability of test results, emergency immunoassay testing introduces unique operational challenges. To mitigate financial burdens, some laboratories arbitrarily extend IQC batch lengths or reduce the number of quality control (QC) samples per analytical run. This study evaluated baseline IQC practices for emergency immunoassays across five laboratories in Suining, China, aiming to provide evidence-based guidance for regional quality improvement.

**Methods:**

Average daily test volumes, external quality assessment (EQA) results, and historical IQC data were retrospectively collected from the five laboratories. Sigma metrics were calculated for each analyte. Concurrently, the “Westgard Sigma Rule with Run Length” nomogram was applied to design individualized, risk-based statistical quality control (RB-SQC) procedures.

**Results:**

All emergency immunoassays, except for serum procalcitonin, were subjected to routine IQC across all five laboratories; notably, certain analytes followed an extended 72-h IQC batch interval. Within individual laboratories, QC rules, the number of QC results per batch, and batch lengths were consistent across analytes but varied between laboratories. Under the current QC scheme, the QC utilization rate (N/M-1) was 0.087 (0.047–0.143), which decreased significantly to 0.020 (0.004–0.133) after implementing RB-SQC procedures (N/M-2; *Z* = 3.154, *P* < 0.05), suggesting that RB-SQC may reduce QC material and reagent consumption while maintaining acceptable patient risk. However, arbitrary adjustments to batch lengths or the number of QC results may lead to elevated or reduced QC utilization rates.

**Conclusions:**

Baseline IQC practices for emergency immunoassays in the surveyed laboratories were inconsistent and lacked a standardized risk-based foundation. We recommend that clinical laboratories implement individualized RB-SQC protocols designed using the Westgard Sigma Rule with Run Length nomogram, and periodically reassess their appropriateness based on updated sigma metrics.

## 1 Introduction

Emergency rapid testing is a cornerstone of acute care medicine, necessitating uninterrupted, 24-h clinical laboratory operations (1). However, uneven distribution of healthcare resources across China has led to pronounced disparities in emergency testing capabilities among medical institutions (1–4). To standardize practices, the Chinese Society of Laboratory Medicine, the Chinese College of Emergency Physicians, and the Emergency Medicine Professional Committee of the Chinese People's Liberation Army jointly issued the *Consensus of Chinese Experts on the Construction and Standardization of Emergency Medical Laboratory Capability* (1). This landmark consensus provides comprehensive framework guidance on resource allocation, assay selection, quality management, and workflow optimization to narrow regional capacity gaps.

Guided by this expert consensus, numerous medical institutions have expanded their emergency immunoassay menus to align with their patient demographics and institutional specialties (2). To guarantee the ongoing clinical validity of these time-sensitive results, laboratories must engage in routine external quality assessment (EQA) schemes and maintain stringent internal quality control (IQC) procedures (1, 5, 6). Traditional regulatory guidelines typically mandate daily IQC for each analyte, requiring at least two levels of control materials (normal and abnormal) per analytical run (5). However, due to the characteristics of emergency immunoassays, such as smaller daily workloads and higher reagent costs, adhering to the regulations or guidelines for IQC procedure run lengths and the number of quality control (QC) results might increase laboratory testing costs. To reduce costs, some laboratories might blindly increase the IQC procedure run lengths and reduce the number of QC results, which may not reduce patient risk to clinically acceptable levels (7–9).

To ensure the accuracy of test results and minimize patient risk, clinical laboratories have introduced risk management concepts from industrial production into quality management (10–12). Initially, clinical laboratories used quality tools such as power function graphs and process control charts to design statistical quality control (SQC) procedures. However, these tools have drawbacks, including complex calculations and difficulties in designing QC frequencies (10–14). To address this issue, Westgard et al. (10) developed a simple and practical new tool for risk-based (RB) SQC procedure design, called the “Westgard Sigma Rule with Run Length,” which combines patient risk parameter MaxE (N_uf_) with classic Westgard sigma rules. By setting MaxE (N_uf_) = 1 and incorporating analyte-specific sigma metrics, this tool derives RB-SQC protocols that align with clinical patient safety thresholds. This framework ensures that the number of unacceptable patient results arising from out-of-control conditions is restricted to a maximum of one per analytical batch, thereby achieving risk-oriented quality control optimization (10).

Clinical Laboratory Quality Control Centers (CLQCCs) were established to regularly inspect and supervise clinical laboratories within their jurisdictions, thereby enhancing service quality and testing capabilities across Chinese provinces and cities. Due to the rising burden of cardiovascular diseases and antibiotic overuse, CLQCCs have increasingly prioritized the quality and performance of emergency immunoassays—including myoglobin (MYO), creatine kinase-MB (CK-MB), high-sensitivity cardiac troponin I (hs-cTnI), B-type natriuretic peptide (BNP), and procalcitonin (PCT) (7, 8). Suining, located in western China, faces limited healthcare resources and pronounced urban–rural disparities (4). This imbalance in healthcare infrastructure contributes to insufficient emergency services in local primary care institutions (1–3). To assess emergency laboratory quality and capacity in the region, this study included five laboratories: two from national secondary hospitals, two from tertiary hospital branches, and one from a national tertiary specialized hospital. All participated in the National Center for Clinical Laboratories (NCCL) EQA programs.

Therefore, this study analyzes the current status of IQC for emergency immunoassays in five clinical laboratories in Suining, China, and designs individualized RB-SQC procedures based on their performance levels to guide laboratories in quality improvement and enhance emergency rapid testing services.

## 2 Material and methods

### 2.1 Materials

This study focused on five emergency immunoassays—MYO, CK-MB, hs-cTnI, BNP, and PCT—across five clinical laboratories (designated A to E) in Suining, China. All analytes were quantified using chemiluminescent immunoassays, with each laboratory employing distinct reagent-instrument combinations from different manufacturers: Laboratory A used the UniCel DxI 800 Access Immunoassay System (Beckman Coulter, USA); Laboratory B used the LA3000 Chemiluminescence System (Sichuan Orienter Biotechnology Co., Ltd., China); Laboratory C used the Mindray CL-6000i Access Immunoassay System (Shenzhen Mindray Bio-Medical Electronics Co., Ltd., China); Laboratory D used the Maccura i3000 Chemiluminescence System (Maccura Biotechnology Co., Ltd., China); Laboratory E used the Abbott i2000SR Chemiluminescence System (Abbott Laboratories, USA).

Except for Laboratory D, which did not implement IQC procedures for PCT testing, all other four laboratories used Lyphochek Specialty Immunoassay Control (Bio-Rad Laboratories Inc., Hercules, CA, USA) for PCT IQC. Among these, Laboratories A and B employed only Level 2, while Laboratories C and E utilized both Level 2 and Level 3. For the cardiac markers—MYO, CK-MB, hs-cTnI, and BNP—all five laboratories consistently used Liquichek Cardiac Markers Plus Control (Bio-Rad Laboratories Inc., Hercules, CA, USA). Laboratories A and B applied only Level 1, whereas Laboratories C, D, and E implemented both Level 1 and Level 2. All QC materials were stored and used strictly in accordance with the manufacturer's product instructions.

Data on average daily test volume and IQC metrics for each analyte were collected from the five laboratories between January and December 2022. IQC data included batch length (*M*), the number of QC results per batch (*N*), out-of-control decision rules, and the coefficient of variation (CV) from 6 months of cumulative in-control data using the same batch of control materials for each analyte (Table 1). Additionally, for the BNP assay, test results from two EQA activities organized by NCCL in 2022 and 2023 were obtained. For the MYO, CK-MB, hs-cTnI, and PCT assays, EQA results from two 2022 assessments were also included. Each NCCL-organized EQA activity included five sample batches per analyte.

**Table 1:** The baseline IQC of emergency immunoassays in five clinical laboratory.

Analytes	Laboratory	Average daily test volume of patient samples	QC rules	*N*	Interval between consecutive IQC runs (days)	*M*	N/M
MYO	A	37	1_3S_/2_2S_/R_4S_/10_χ_	1	1	37	0.027
	B	6	1_3S_/2_2S_	1	3	19	0.053
	C	23	1_3S_/2_2S_	2	1	23	0.087
	D	2	1_3S_/2_2S_	2	1	2	0.917
	E	21	1_3S_/2_2S_/R_4S_	2	1	21	0.096
CK-MB	A	37	1_3S_/2_2S_/R_4S_/10_χ_	1	1	37	0.027
	B	6	1_3S_/2_2S_	1	3	19	0.053
	C	23	1_3S_/2_2S_	2	1	23	0.087
	D	2	1_3S_/2_2S_	2	1	2	0.917
	E	21	1_3S_/2_2S_/R_4S_	2	1	21	0.096
hs-cTnI	A	37	1_3S_/2_2S_/R_4S_/10_χ_	1	1	37	0.027
	B	6	1_3S_/2_2S_	1	3	19	0.053
	C	23	1_3S_/2_2S_	2	1	23	0.087
	D	11	1_3S_/2_2S_	2	1	11	0.189
	E	21	1_3S_/2_2S_/R_4S_	2	1	21	0.096
BNP	A	30	1_3S_/2_2S_/R_4S_/10_χ_	1	1	30	0.034
	B	6	1_3S_/2_2S_	1	3	18	0.056
	C	23	1_3S_/2_2S_	2	1	23	0.087
	D	10	1_3S_/2_2S_	2	1	10	0.199
	E	6	1_3S_/2_2S_/R_4S_	2	1	6	0.326
PCT	A	25	1_3S_/2_2S_/R_4S_/10_χ_	1	1	25	0.040
	B	6	1_3S_/2_2S_	1	3	18	0.056
	C	21	1_3S_/2_2S_	2	1	21	0.096
	D	9	N/A	N/A	N/A	N/A	N/A
	E	10	1_3S_/2_2S_/R_4S_	2	1	10	0.206

IQC, internal quality control; QC rules, quality control rules; N, number of quality control results; M, batch length, and expressed as the average number of patient samples per run of one internal quality control procedure; N/M, QC utilization rate; N/A, not applicable.

### 2.2 Methods

#### 2.2.1 Survey of baseline IQC practices and daily test volume data

We systematically surveyed the baseline IQC practices for emergency immunoassays across the five laboratories, focusing on established QC decision rules, the number of QC results per batch (*N*), and the interval between consecutive IQC runs. Additionally, the average daily test volume of patient samples was recorded for each analyte. The batch length (*M*) for baseline IQC practices was calculated as: *M* = interval between consecutive IQC runs (days) × average daily test volume of patient samples.

#### 2.2.2 Evaluation of bias

All five laboratories participated in EQA activities organized by the NCCL for each analyte. The laboratories tested EQA samples and submitted results according to NCCL requirements. The NCCL grouped laboratory-reported results by reagent manufacturer, then applied ISO 13528 to calculate the robust mean for each group, which served as the target value for EQA samples. In this study, the reported results of each analyte from the five laboratories were categorized into different reagent groups. Target values for the EQA samples were obtained from the NCCL's official website (https://www.nccl.org.cn/mainCn). The absolute percentage difference between laboratory results and target values was calculated. The mean absolute percentage difference across 10 EQA samples was used to estimate the average bias for each emergency immunoassay (Table 2) (15, 16). The formula for bias was defined as follows:


$$\begin{array}{l} {\text{Bias}}_{\text{LOT}i}=\frac{\left|\text{test }{\text{result}}_{\text{LOT}i}-\text{target }{\text{value}}_{\text{LOT}i}\right|}{\text{target }{\text{value}}_{\text{LOT}i}},\;i\;=\;1,\;2,\;3\;\ldots \text{ n}\\ \text{ Bias}=\frac{{\text{Bias}}_{\text{LOT1}}+{\text{Bias}}_{\text{LOT2}}+\cdots +{\text{Bias}}_{\text{LOT}n}}{n} \end{array}$$

**Table 2:** The Sigma metrics values for emergency immunoassays in five clinical laboratories.

Analytes	Laboratory	TEa(%)	CV (%)			Bias (%)	σ	QGI	Quality improvement measures
		CV_1_	CV_2_	RMS CV					
MYO	A	30	6.30	N/A	N/A	3.29	4.24	0.35	Imprecision
	B	30	5.28	N/A	N/A	1.31	5.43	0.17	Imprecision
	C	30	6.42	6.16	6.29	2.29	4.40	0.24	Imprecision
	D	30	8.49	8.77	8.63	2.84	3.15	0.22	Imprecision
	E	30	5.01	4.93	4.98	2.41	5.54	0.32	Imprecision
CK-MB	A	30	6.10	N/A	N/A	4.84	4.12	0.53	Imprecision
	B	30	4.23	N/A	N/A	4.60	6.00	0.73	N/A
	C	30	6.51	6.32	6.42	4.07	4.04	0.42	Imprecision
	D	30	6.56	7.10	6.84	4.01	3.80	0.39	Imprecision
	E	30	6.03	5.42	5.73	4.46	4.46	0.52	Imprecision
hs-cTnI	A	30	4.80	N/A	N/A	5.51	5.10	0.76	Imprecision
	B	30	4.28	N/A	N/A	3.68	6.15	0.57	N/A
	C	30	6.17	5.99	6.08	3.03	4.44	0.33	Imprecision
	D	30	8.56	8.77	8.67	11.67	2.12	0.90	Imprecision and Bias
	E	30	5.43	5.86	5.65	4.41	4.53	0.52	Imprecision
BNP	A	30	3.90	N/A	N/A	4.74	6.48	0.81	N/A
	B	30	4.11	N/A	N/A	3.17	6.53	0.51	N/A
	C	30	6.92	6.73	6.83	1.47	4.18	0.14	Imprecision
	D	30	5.37	4.73	5.06	10.03	3.95	1.32	Bias
	E	30	6.44	3.63	5.23	9.98	3.83	1.27	Bias
PCT	A	30	4.50	N/A	N/A	4.32	5.71	0.64	Imprecision
	B	30	6.25	N/A	N/A	3.71	4.21	0.40	Imprecision
	C	30	5.94	7.73	6.89	5.55	3.55	0.54	Imprecision
	D	30	N/A	N/A	N/A	3.25	N/A	N/A	Imprecision
	E	30	6.40	4.93	5.71	4.43	4.48	0.52	Imprecision

TEa, total allowable error, they were from the NCCL EQA performance specifications for 2022–2025; CV, cumulative coefficient of variation; CV_1_ and CV_2_, the cumulative coefficient of variation of quality control substance at different concentration levels; RMS CV, root mean square coefficient of variation; σ, sigma metrics; QGI, Quality Goal Index; N/A, not applicable.

#### 2.2.3 Evaluation of precision

The CV from 6 months of cumulative in-control data for each analyte, using the same control material batch, was collected to estimate imprecision (Table 2). Root mean square CV (RMS CV%) was used to estimate imprecision for two control levels. CV_1_ and CV_2_ represent cumulative CVs at two concentration levels for each emergency immunoassay (12). The RMS CV% was calculated using the following formula:


$$\begin{array}{c} \text{RMS CV}\%=\sqrt{\frac{\left({\text{CV}}_{1}^{2}+{\text{CV}}_{2}^{2}\right)}{2\;}} \end{array}$$

#### 2.2.4 Sigma metrics calculation

The selection of appropriate Total Allowable Error (TEa) thresholds is critical for accurate sigma metric calculation. In accordance with the updated Clinical Laboratory Improvement Amendments (CLIA) proficiency testing requirements effective January 2025, TEa specifications for BNP, CK-MB, and hs-cTnI are defined as Target Value ± 30%, Target Value ± 25% or ± 3 ng/ml (whichever is greater), and Target Value ± 25% or ± 0.9 ng/ml (whichever is greater), respectively (17). However, CLIA 2025 does not establish TEa limits for MYO and PCT (17). In China, NCCL routinely sets performance specifications for all analytes in its EQA programs. For the period 2022–2025, NCCL applied a uniform TEa criterion of Target Value ± 30% to MYO, CK-MB, hs-cTnI, BNP, and PCT. Given that NCCL EQA results are formally integrated into the annual performance evaluation of clinical laboratories in China, these criteria are widely adopted as the operational TEa standards in routine quality management. Therefore, the TEa values used in this study for all five analytes are derived from the NCCL EQA performance specifications for 2022–2025.

Sigma metrics (σ) for each analyte were calculated using the following formula, where *n* represents the number of control material concentration levels.


$$\begin{array}{l} \sigma =\left(\text{TEa}-\text{Bias}\right)/\text{RMS CV }\left(n=2\right)\text{ or}\\ \sigma =\left(\text{TEa}-\text{Bias}\right)/{\text{CV}}_{1}\left(n=\;1\right) \end{array}$$

Additionally, the Quality Goal Index (QGI) was calculated for each analyte to guide the prioritization of quality improvement strategies (14, 16, 18–20). QGI quantifies whether an analyte's performance is primarily limited by imprecision or bias, calculated as QGI = Bias/(1.50 × RMS CV) (*n* = 2) or QGI = Bias/(1.50 × *CV*_1_) (*n* = 1), where *n* represents the number of QC concentration levels. Based on QGI values, improvement priorities were defined as follows: QGI <0.80, prioritize imprecision reduction; 0.80 ≤ QGI ≤ 1.20, both imprecision and bias require improvement; QGI > 1.20, prioritize bias correction (14, 16, 18–20).

#### 2.2.5 Selection of RB-SQC procedures

Based on the calculated Sigma metrics for each analyte, individualized risk-based statistical quality control (RB-SQC) procedures were designed utilizing the “Westgard Sigma Rule with Run Length” framework. These tailored procedures defined the optimal combination of QC decision rules, QC sample size (*N*), and maximum allowable batch length (*M*) (10, 16, 19). For σ ≥ 6, 1_3S_ with *N* = 2 and *M* = 1,000; for 5 ≤ σ <6, 1_3S_/2_2S_/R_4S_ multi-rules with *N* = 2 and *M* = 450; for 4 ≤ σ <5, 1_3S_/2_2S_/R_4S_/4_1S_ multi-rules with *N* = 4 and *M* = 200; for σ <4, 1_3S_/2_2S_/R_4S_/4_1S_/6_χ_ multi-rules with *N* = 6 and *M* = 45, necessitating more frequent QC activities (10, 16, 19). After designing the RB-SQC procedures, the interval between consecutive RB-SQC runs (days) was calculated for each analyte as: interval between consecutive RB-SQC runs (days) = *M*/Average daily test volume of patient samples.

#### 2.2.6 Comparison of the utilization rates of quality control results

The QC utilization rate (N/M), defined as the number of QC measurements per patient sample (N/M = QC sample size / Maximum allowable batch length), was calculated for each assay. The current rate for each laboratory's QC scheme was denoted as N/M-1, while that after implementing the RB-SQC program was denoted as N/M-2.

In some laboratories, the average daily test volume for certain emergency immunoassays was low, resulting in RB-SQC batch lengths exceeding 24 h. As traditional regulatory guidelines typically mandate daily QC, the batch length was adjusted to 24 h while keeping the QC rules unchanged. When the number of QC results per batch (*N*) was set to 1 under this adjusted schedule, the utilization rate was denoted as N/M-3; when *N* was set to 2, it was denoted as N/M-4. These rates were calculated as: N/M-3 = 1 / Average daily test volume of patient samples; N/M-4 = 2 / Average daily test volume of patient samples.

**Figure 1:**
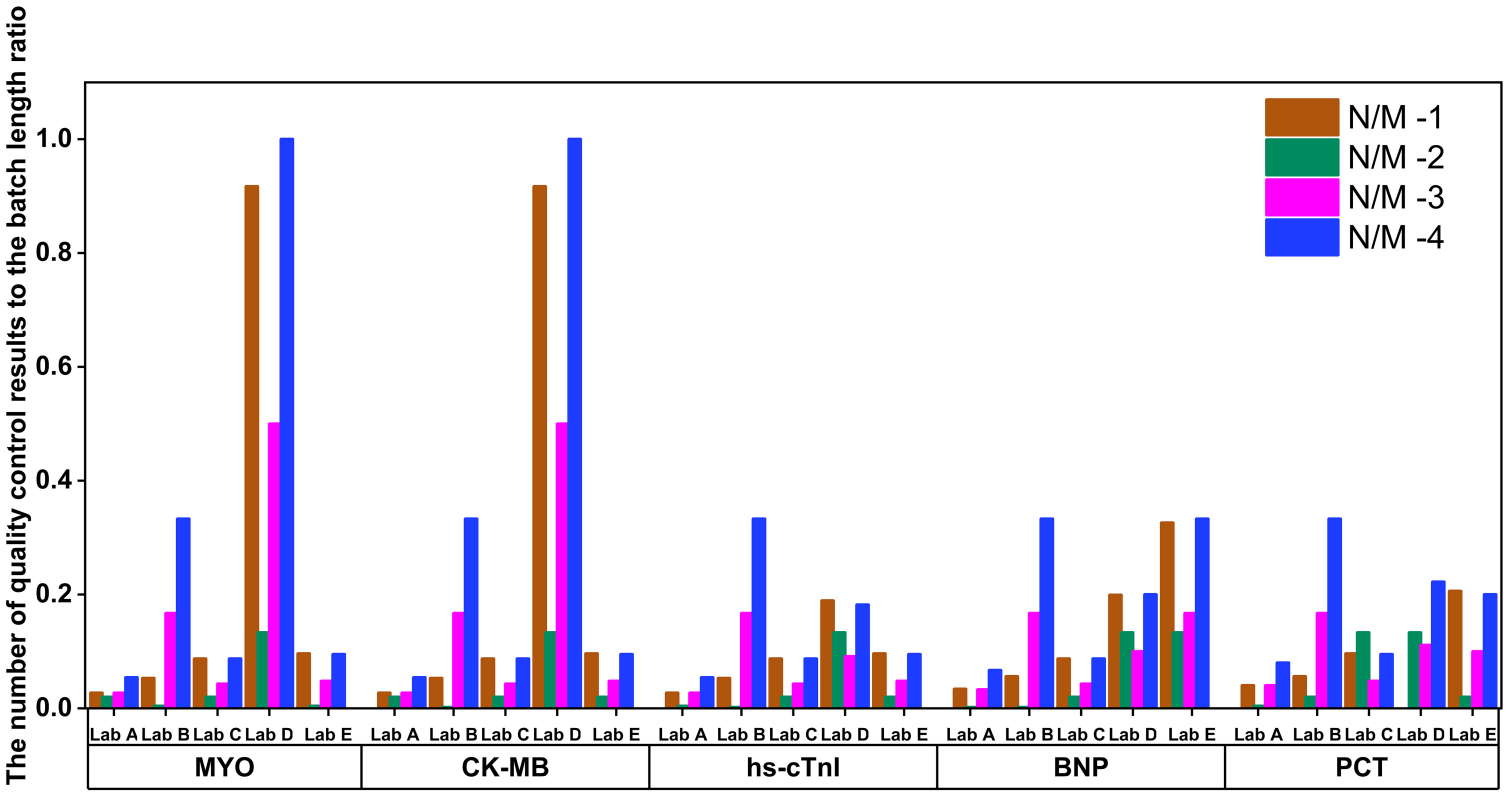
Comparison of QC utilization rates under different quality control schemes. QC, quality control; N/M-1 represents the utilization rate of the current quality control scheme; N/M-2 represents the utilization rate after implementing the risk-based statistical quality control procedures; N/M-3 represents the utilization rate of the risk-based statistical quality control procedures with one quality control result per day; N/M-4 represents the utilization rate of the risk-based statistical quality control procedures with two quality control results per day.

The QC utilization rate is inversely related to the number of patient samples tested between consecutive QC runs and directly related to *N*. A lower N/M ratio reflects a concomitant reduction in the consumption of QC materials, dedicated reagents, and technician labor per patient sample tested, thereby minimizing the unit analytical cost. The N/M values for each analyte were compared across the four protocols (Figure 1).

### 2.3 Statistical analysis

Data analysis employed Microsoft Excel (Microsoft, Redmond, WA, USA) and IBM SPSS Statistics 19.0, while OriginLab Origin 2021 was used for graphing. Given the limited sample size and potential skewness in QC utilization rates, we report the median (25th−75th percentile) for each group under the four QC schemes. The Mann–Whitney U test was applied for intergroup comparisons, with statistical significance set at *P* < 0.05.

## 3 Results

### 3.1 The baseline IQC of emergency immunoassays in five laboratories

The baseline IQC practices for emergency immunoassays across the five laboratories are summarized in Table 1. Laboratories A, B, C, and E performed IQC for all analytes, whereas Laboratory D performed IQC only for MYO, CK-MB, hs-cTnI, and BNP. All laboratories applied at least the 1_3S_/2_2S_ rules to determine whether QC results were out of control. Within each laboratory, QC rules, the number of QC results per batch, and batch lengths were consistent across all analytes. In comparing laboratories, all except Laboratory B—which conducted IQC every 72 h—performed daily IQC procedures. Laboratories A and B applied a consistent number of QC results per batch across analytes, as did Laboratories C, D, and E. The QC utilization rates (N/M) ranged from 0.027 to 0.917, except for the PCT analyte in Laboratory D.

### 3.2 Sigma metrics values of emergency immunoassays in five laboratories

Except for the hs-cTnI assay in Laboratory D, which demonstrated a Sigma value of 2.12, and the PCT assay, which could not be evaluated due to insufficient data, all remaining assays met or exceeded the 3σ threshold across the five laboratories. Notably, only CK-MB, hs-cTnI, and BNP achieved superior 6σ performance, and only in certain laboratories (Table 2). QGI analysis revealed the following priorities: simultaneous improvement of imprecision and bias was needed for the hs-cTnI assay in Laboratory D; bias improvement was prioritized for the BNP assays in Laboratories D and E; and for the remaining immunoassays, imprecision improvement was the main target, particularly for the PCT assay in Laboratory D.

### 3.3 RB-SQC procedures for each emergency immunoassay in five laboratories

Table 3 presents the RB-SQC procedures designed for each emergency immunoassay. Based on each laboratory's average daily test volume, the interval for one IQC cycle per analyte ranged from 2.14 to 166.67 days. The QC utilization rates (N/M) ranged from 0.002 to 0.133.

**Table 3:** The RB-SQC procedures for each emergency immunoassay in five clinical laboratories.

Analytes	Laboratory	Average daily test volume of patient samples	σ	QC rules	*N*	*M*	Interval between consecutive RB-SQC runs (days)	N/M
MYO	A	37	4.24	1_3S_/2_2S_/R_4S_/4_1S_	4	200	5.41	0.020
	B	6	5.43	1_3S_/2_2S_/R_4S_	2	450	75.00	0.004
	C	23	4.40	1_3S_/2_2S_/R_4S_/4_1S_	4	200	8.70	0.020
	D	2	3.15	1_3S_/2_2S_/R_4S_/4_1S_/6_χ_	6	45	22.50	0.133
	E	21	5.54	1_3S_/2_2S_/R_4S_	2	450	21.43	0.004
CK-MB	A	37	4.12	1_3S_/2_2S_/R_4S_/4_1S_	4	200	5.41	0.020
	B	6	6.00	1_3S_	2	1,000	166.67	0.002
	C	23	4.04	1_3S_/2_2S_/R_4S_/4_1S_	4	200	8.70	0.020
	D	2	3.80	1_3S_/2_2S_/R_4S_/4_1S_/6_χ_	6	45	22.50	0.133
	E	21	4.46	1_3S_/2_2S_/R_4S_/4_1S_	4	200	9.52	0.020
hs-cTnI	A	37	5.10	1_3S_/2_2S_/R_4S_	2	450	12.16	0.004
	B	6	6.15	1_3S_	2	1,000	166.67	0.002
	C	23	4.44	1_3S_/2_2S_/R_4S_/4_1S_	4	200	8.70	0.020
	D	11	2.12	1_3S_/2_2S_/R_4S_/4_1S_/6_χ_	6	45	4.09	0.133
	E	21	4.53	1_3S_/2_2S_/R_4S_/4_1S_	4	200	9.52	0.020
BNP	A	30	6.48	1_3S_	2	1,000	33.33	0.002
	B	6	6.53	1_3S_	2	1,000	166.67	0.002
	C	23	4.18	1_3S_/2_2S_/R_4S_/4_1S_	4	200	8.70	0.020
	D	10	3.95	1_3S_/2_2S_/R_4S_/4_1S_/6_χ_	6	45	4.50	0.133
	E	6	3.83	1_3S_/2_2S_/R_4S_/4_1S_/6_χ_	6	45	7.50	0.133
PCT	A	25	5.71	1_3S_/2_2S_/R_4S_	2	450	18.00	0.004
	B	6	4.21	1_3S_/2_2S_/R_4S_/4_1S_	4	200	33.33	0.020
	C	21	3.55	1_3S_/2_2S_/R_4S_/4_1S_/6_χ_	6	45	2.14	0.133
	D	9	N/A	1_3S_/2_2S_/R_4S_/4_1S_/${\text{6}}_{\text{χ}}^{1}$	6^1^	45^1^	5.00	0.133
	E	10	4.48	1_3S_/2_2S_/R_4S_/4_1S_	4	200	20.00	0.020

RB-SQC, risk-based statistical quality control; σ, sigma metrics; QC rules, quality control rules; N, number of quality control results; M, batch length, and expressed as statistical quality control procedure allows maximum run size of patient sample; N/M, quality control results utilization rate; N/A, not applicable.

^1^Since no IQC data were available for PCT in Laboratory D, sigma metrics could not be calculated, and the analytical performance could not be accurately assessed. Therefore, the most conservative RB-SQC procedure was applied: 13S/22S/R4S/41S /6$ multi-rules with N = 6 and M = 45, necessitating more frequent QC activities.

### 3.4 Comparison of QC utilization rates for different QC schemes

Figure 1 summarizes QC utilization rates for emergency immunoassays across the five laboratories under four different QC schemes. The current QC scheme's utilization rate (N/M-1) was 0.087 (0.047–0.143), which significantly decreased to 0.020 (0.004–0.133) following implementation of RB-SQC procedures (N/M-2). N/M-2 showed statistically significant differences from N/M-1 (*Z* = 3.154, *P* < 0.05). When SQC procedures were adjusted to run every 24 h—reflecting routine clinical practice—the utilization rate was 0.048 (0.043–0.167) with one QC result per batch (N/M-3), and increased to 0.095 (0.087–0.333) with two QC results per batch (N/M-4). Both N/M-3 and N/M-4 showed statistically significant differences from N/M-2 (*Z* = 3.782 and 4.329, respectively; both *P* < 0.05).

## 4 Discussion

The investigation showed that IQC was performed for MYO, CK-MB, hs-cTnI, and BNP in all five laboratories, while PCT was tested only in laboratories A, B, C, and E. This suggests inadequate IQC implementation for some emergency immunoassays in select laboratories. Analysis of Laboratory D's omission of PCT IQC reveals that MYO and CK-MB, despite having lower daily test volumes, were still subject to the same IQC frequency as hs-cTnI and BNP (i.e., testing two QC levels every 24 h). These findings suggest that IQC implementation may be influenced by factors beyond test volume (21). MYO, CK-MB, hs-cTnI, and BNP frequently shared QC materials, while PCT required separate procurement and individual testing. This may increase staff workload. Additionally, PCT reagents were relatively costly per test. Adhering to guidelines requiring two QC levels tested every 24 h could substantially raise testing costs (5). Therefore, IQC procedures may be influenced by factors including average daily test volume, reagent and QC material costs, and staff workload (21–23).

Wheeler et al. (5, 22) reported that most countries/regions have explicit IQC requirements, including testing at least two QC levels (normal and abnormal) daily. However, ISO 15189:2022 now mandates that batch length and the number of QC samples per batch be determined based on method robustness and patient risk from erroneous results (24). This study observed substantial variation in average daily test volume across analytes; for example, in Laboratory D, the volume for hs-cTnI was 5.5-fold higher than that for MYO. Notably, IQC rules, QC frequency, and batch lengths remained uniform within each laboratory (Table 1). This suggests that IQC procedures were not individualized based on analyte performance, consistent with findings by Tingting Li (8). Arbitrarily increasing or decreasing QC frequency or batch length can result in over-control or under-control, potentially impacting both laboratory cost-efficiency and patient safety (5, 25, 26). Consequently, the “Westgard Sigma Rule with Run Length” has been increasingly applied to design SQC procedures in clinical laboratories (10, 16, 19).

Analytical performance was typically evaluated using σ which is computed from TEa, bias, and imprecision (CV)—collectively influencing analyte sigma metric calculation. TEa denotes the quality goal established by clinical laboratories in accordance with clinical needs; once set, this goal does not further influence sigma metric evaluation or RB-SQC procedure design. CV embodies the inherent imprecision of the laboratory detection system, with the cumulative coefficient of variation (CV) of in-control IQC data commonly serving as an estimator of analyte imprecision. Currently, for the IQC procedures of emergency immunoassays, most clinical laboratories utilize QC materials characterized by extended stability and a vial-to-vial variability that is lower than the inherent variation of the analytical system. However, due to subtle discrepancies in the antibodies or antigens utilized across different reagent lots from the same manufacturer, variations in binding kinetics during solid-phase coating, and the potential lack of commutability inherent to commercial matrices, the confluence of these factors may induce systematic shifts in the measurement results of the same lot of long-stability QC material when transitioning between different reagent lots (27). Consequently, directly employing the cumulative coefficient of variation (CV) derived from 6 months of long-term in-control IQC data may fail to objectively reflect the true imprecision of emergency immunoassays. To ensure an objective evaluation of analyte imprecision, clinical laboratories are advised to strengthen stability assessments for both long- and short-stability QC materials (27). Furthermore, laboratories should perform robust lot-to-lot validation between new and old reagent lots using commutable, fresh patient samples (27). This approach evaluates result consistency across different reagent configurations and verifies whether the shifting trends of QC data parallel those observed in patient samples. If necessary, a pooled or averaged imprecision model may be adopted to achieve a more reliable estimation of analyte imprecision. For the five emergency immunoassays evaluated (MYO, CK-MB, hs-cTnI, PCT, and BNP), universally accepted reference materials and reference methods are currently unavailable. Moreover, immunoassay kits from different manufacturers employ distinct antibody clones and epitope specificities, leading to systematic inter-platform discrepancies that render cross-system result comparability inherently limited. Therefore, it should be acknowledged that the bias estimates in this study were derived from EQA peer-group target values rather than from reference methods or certified reference materials (28). Consequently, EQA programs can only stratify laboratories by reagent manufacturer, and peer-group means—rather than trueness-based targets—serve as the de facto bias estimators, which may not objectively evaluate analyte bias and could consequently affect sigma metric calculations and RB-SQC procedure design (28, 29). Additionally, bias estimation in this study relied on only 10 EQA samples per analyte per cycle. Although this sample size provides a preliminary estimate of systematic deviation, it may be insufficient for robust bias characterization, particularly for analytes with high between-run variability. To address these limitations, efforts should be made to develop internationally recognized reference materials and reference methods for emergency immunoassay analytes, or to employ an accredited reference measurement service to facilitate trueness transfer to this designated system, thereby improving result comparability (28, 29). Future EQA programs should consider adopting commutable materials, such as fresh-frozen human serum pools, as assignable targets to enable objective, method-independent bias assessment and more reliable sigma metric calculation (28, 29).

As indicated in Table 2, only CK-MB, hs-cTnI, and BNP reached 6σ performance in selected laboratories. Thus, laboratories should prioritize quality improvement efforts for analytes with σ values below 6. This study employed QGI analysis to inform laboratory quality improvement strategies (20). Findings indicated that for most analytes with σ <6, imprecision improvement should be prioritized—likely due to low daily test volumes and large packaging sizes of emergency immunoassay kits. Lower daily workloads result in prolonged reagent use and extended on-instrument time. This may compromise reagent stability and lead to increased imprecision in analyte measurements (9, 18). Therefore, for low-volume assays, special consideration should be given to post-opening reagent stability during performance evaluations. Continued use of the Clinical and Laboratory Standards Institute (CLSI) EP15-A2/A3 protocol for imprecision evaluation may be inadequate in this context. Instead, the CLSI EP5-A2 guideline is recommended, which entails single-batch daily testing over 20 consecutive days (30). This longer evaluation period more accurately reflects within-laboratory imprecision under routine operating conditions than do shorter protocols.

Individualized RB-SQC procedures were designed based on sigma metrics (Table 3), revealing significant variability among analytes both within and across laboratories. Comparison of the N/M ratios (number of QC results to batch length) showed that PCT in Laboratories C and D exhibited lower N/M-1 values, indicating under-control and inadequate risk reduction—likely attributable to poorer analytical performance or the prior absence of IQC for PCT. In contrast, other immunoassays demonstrated higher N/M-1 values, suggesting over-control. As illustrated in Figure 1, arbitrary adjustments to batch lengths or QC frequency led to either over- or under-control (N/M-3 or N/M-4). This underscores the importance of tailoring RB-SQC procedures to analyte-specific analytical performance (10, 16, 19).

In clinical laboratories, the total cost of analyte testing is primarily comprised of five major categories: reagents and consumables, equipmentmaintenance, personnel labor, facility operations, and quality management (31). Within this framework, multiple elements of the IQC program—such as QC decision rules, the number of QC samples per analytical run (*N*), the maximum allowable batch length (*M*), corrective actions triggered by QC failure, calibration frequency, lot-to-lot reagent verification, and dilution of high-concentration specimens—exert a substantial influence on the overall quality management cost. This study focuses specifically on RB-SQC strategies, aiming to preliminarily explore their impact on the analytical cost associated with emergency immunoassays. As illustrated in Figure 1, implementing RB-SQC protocols significantly reduces the N/M ratio for most analytes. As previously defined, a lower N/M ratio directly translates to decreased consumption of QC materials, dedicated reagents, and technician labor per patient sample tested. This targeted reduction in redundant QC activities consequently lowers the unit analytical cost, while enabling more efficient resource allocation toward analytes with inherently lower analytical performance that require more frequent QC monitoring.

However, when stratified by the average daily test volume of patient samples, RB-SQC procedures could be scheduled every 2.14 to 166.67 days while still maintaining clinically acceptable risk—contrasting with the routine practice of daily testing of two QC levels prior to patient testing (5, 7, 8). If IQC frequency is strictly designed based on maximum run length for analytes with low daily test volumes, procedures may only be required a few times per year. This could hinder the laboratory's ability to promptly detect analytical errors in the testing process. Therefore, once RB-SQC procedures are designed, their appropriateness should be evaluated based on the analyte's intended clinical application. Gidske et al. (9) developed a scoring system to guide IQC frequency for point-of-care testing devices in primary care, considering analyte clinical importance, device type, and ease of use. However, due to the strong link between timely and accurate emergency immunoassay results and patient safety (1–3, 7, 32), all emergency immunoassays in the five laboratories scored 7–9 points using this system (9). Accordingly, IQC should be performed at least weekly—and additionally upon new reagent lot introduction, unexpected results, suspected analytical errors, or post-maintenance—to minimize analytical risk (9). This suggests limitations in applying RB-SQC procedures in smaller laboratories or for analytes with low testing frequency. Conversely, patient-based real-time quality control (PBRTQC) has demonstrated effectiveness in continuously monitoring analytical performance and promptly detecting system deviations (33–36). Therefore, clinical laboratories are encouraged to integrate the Westgard Sigma Rule with Run Length, PBRTQC, and the Gidske scoring system to develop robust, evidence-based IQC protocols.

Crucially, the routine clinical practice of synchronized internal quality control (IQC)—where all analytes on a shared platform are tested simultaneously before daily patient testing—may lack a solid theoretical foundation. The findings of this regional investigation revealed that key cardiac and infectious disease biomarkers, including MYO, CK-MB, hs-cTnI, PCT, and BNP, were invariably monitored on the same integrated immunoassay analyzer across all five participating facilities. Because these individual assays inherently rely on identical hardware subsystems—such as the shared sample pipetting assembly, reagent dispensing arm, incubation chambers, signal detection optics, and washing stations—the analytical validation of any single analyte effectively monitors the operational integrity of these shared internal components. Within the established shelf life of a reagent kit, given stable calibration curves and parameters, the mathematical probability of an isolated out-of-control event is exceedingly low if alternative assays on the same platform demonstrate stable in-control performance. Conversely, systematic malfunctions within the shared fluidic or optical systems would manifest as concurrent deviations across multiple analytes. Based on theoretical analysis of systemic interdependence, the concept of a staggered IQC scheduling strategy. By strategically distributing QC measurements of different analytes across multiple distinct time points within a 24-h cycle, this approach may compress the temporal windows between sequential control events and enhance the dynamic detection of systematic errors. For example, with the five analytes in this study, testing QC materials at two concentration levels yields 10 QC results; under a staggered IQC scheduling strategy, a QC result becomes available every 2–3 h for monitoring system stability. The potential of this dynamic, distributed monitoring framework lies in its ability to increase the frequency of hardware integrity checks without incurring additional reagent or material costs, thereby theoretically improving error detection probability. It should be emphasized that this strategy has not been empirically evaluated in this study; its clinical feasibility, operational practicality, and performance benefits require validation through future experimental and field-based studies.

Nevertheless, implementing such a sophisticated staggered scheduling model under traditional manual operational workflows presents tangible logistical challenges for laboratory staff. To overcome these operational bottlenecks, fully automated IQC systems have recently been integrated into next-generation clinical laboratory quality management frameworks. In these optimized configurations, operators are only required to aliquot, barcode, and hermetically seal QC materials prior to loading them into the analyzer's integrated refrigeration module. Upon daily system initialization, the platform autonomously retrieves, thaws, homogenizes, and delivers the precise control volumes to the analytical unit via automated tracks, followed by automatic resealing and archival storage. Such pre-programmed digital workflows eliminate manual pipetting variability while enabling highly customized, analyte-specific execution of QC rules, measurement counts, and temporal frequencies (37). Because none of the five standard clinical laboratories evaluated in this study were equipped with these advanced automated IQC platforms, the empirical performance and precise error-detection kinetics of the proposed staggered strategy could not be validated herein. Evaluating the clinical efficacy and cost-benefit ratios of this automated, staggered quality control framework remains a primary objective of our future multi-center research initiatives.

This study has several limitations. First, the QC materials selected by the laboratories may not cover the Analytical Measurement Range of emergency immunoassays in some settings, potentially preventing laboratories from monitoring the quality of high- or low-concentration samples and thereby increasing analytical risk (38, 39). Additionally, the use of a single QC concentration level in some laboratories precludes objective assessment of imprecision at different clinical decision limits from QC data, which may compromise the evaluation of sigma metrics. Nevertheless, the proposed approach still offers value in guiding quality improvement for emergency immunoassays. Second, as the average daily test volume per immunoassay constitutes sensitive data, the reported figures may not accurately reflect the actual values. This could impact the assessment of QC utilization rates but is unlikely to affect RB-SQC design or comparative analyses. Third, for some analytes, RB-SQC procedures required 4_1S_/6χ rules, which may increase false rejection rates and laboratory costs, potentially discouraging implementation. It should be recognized, however, that only when analytical performance is effectively improved can patient risk and laboratory costs be further reduced. Therefore, in the subsequent quality inspection activities, we will focus on the implementation of laboratory quality improvement measures and the re-evaluation of analytical performance. Fourth, although the data analyzed in this study were primarily collected in 2022, the temporal gap between data acquisition and manuscript submission (2026) means that these figures may not perfectly reflect the present analytical performance of the five participating laboratories, especially considering that laboratory quality management has likely advanced over this period. While the core RB-SQC methodology, being derived from analytical performance characteristics and failure mode analysis, remains fully valid regardless of data vintage, the temporal distance warrants caution when extrapolating these results to present-day practice. To address this limitation, future multi-center investigations will incorporate more recent data to track longitudinal quality trends.? Lastly, this study focused on only five laboratories, indicating the need for broader investigations to support nationwide quality improvement. Future QC activities by the CLQCCs will address these gaps to comprehensively enhance the service quality and capabilities of emergency laboratory testing in the region.

## 5 Conclusion

The accuracy and timeliness of emergency test results are critical for ensuring that patients receive effective treatment within narrow therapeutic window. Therefore, assessing the baseline state of IQC and guiding laboratories in quality improvement holds substantial clinical significance. This study demonstrated that baseline IQC practices for emergency immunoassays in the surveyed laboratories were inconsistent and lack a risk-based foundation. Implementing individualized RB-SQC protocols designed via the Westgard Sigma Rule with Run Length framework can effectively optimize QC material consumption while maintaining acceptable patient risk. Therefore, clinical laboratories are advised to adopt such performance-based, risk-tailored QC strategies and periodically review their suitability to ensure ongoing analytical quality.

## Data Availability

The raw data supporting the conclusions of this article will be made available by the authors, without undue reservation.
